# Soil Particle Heterogeneity Affects the Growth of a Rhizomatous Wetland Plant

**DOI:** 10.1371/journal.pone.0069836

**Published:** 2013-07-30

**Authors:** Lin Huang, Bi-Cheng Dong, Wei Xue, Yi-Ke Peng, Ming-Xiang Zhang, Fei-Hai Yu

**Affiliations:** School of Nature Conservation, Beijing Forestry University, Beijing, China; DOE Pacific Northwest National Laboratory, United States of America

## Abstract

Soil is commonly composed of particles of different sizes, and soil particle size may greatly affect the growth of plants because it affects soil physical and chemical properties. However, no study has tested the effects of soil particle heterogeneity on the growth of clonal plants. We conducted a greenhouse experiment in which individual ramets of the wetland plant *Bolboschoenus planiculmis* were grown in three homogeneous soil treatments with uniformly sized quartz particles (small: 0.75 mm, medium: 1.5 mm, or large: 3 mm), one homogeneous treatment with an even mixture of large and medium particles, and two heterogeneous treatments consisting of 16 or 4 patches of large and medium particles. Biomass, ramet number, rhizome length and spacer length were significantly greater in the treatment with only medium particles than in the one with only large particles. Biomass, ramet number, rhizome length and tuber number in the patchy treatments were greater in patches of medium than of large particles; this difference was more pronounced when patches were small than when they were large. Soil particle size and soil particle heterogeneity can greatly affect the growth of clonal plants. Thus, studies to test the effects of soil heterogeneity on clonal plants should distinguish the effects of nutrient heterogeneity from those of particle heterogeneity.

## Introduction

Spatial heterogeneity is an important feature of natural habitats [1]–[5], and plays major roles in shaping plant growth, species interactions, community structure and ecosystem functioning [6]–[15]. Heterogeneity occurs in various spatial scales [2], [3], [5], and the scale of heterogeneity is important because ecological processes that function at one spatial scale may not do so at other scales [16]–[21].

Clonal plants are very abundant in nature and often experience spatial heterogeneity in such a way that connected ramets of the same clone experience contrasting environmental conditions [22]–[24]. Many studies have addressed how spatial heterogeneity in essential resources such as light, soil water and mineral nutrients affects the growth of clonal plants, and these studies generally show that clonal plants can respond to heterogeneity in ways that increase their performance and likely fitness [7], [19]–[20], [25]–[29]. Moreover, clonal plants can respond differently to heterogeneity of soil and light at different spatial scales [18]–[21], [30].

Particle size is an important feature of soil [31]–[34], and it can affect soil physical and chemical properties such as structure, oxygen concentration and water content [33], [35]–[38]. Studies testing the effects of soil nutrient heterogeneity on the performance of clonal plants sometimes compare the growth in substrate with patches of fertile and infertile soil to the growth in a homogeneous mixture of the same amounts of fertile and infertile soil [6], [20]–[21], [39]–[44]. However, fertile and infertile soils generally differ in the size composition of particles, meaning that results of these studies might be partly due to effects of particle size rather than nutrient availability. To test the effects of soil nutrient heterogeneity, therefore, it would be good to create nutrient-rich and nutrient-poor patches with the same type of soil substrate but supplied with different amounts of nutrients [11], [27], [45]–[47]. On the other hand, it would be useful to test the effects of particle size separately. However, no published study appears to have tested the effects of soil particle heterogeneity on the growth of clonal plants.

To test the effects of soil particle heterogeneity on clonal growth, we conducted a greenhouse experiment in which we grew individual ramets of the rhizomatous, wetland plant *Bolboschoenus planiculmis* in three homogeneous soils each containing only one size class of quartz particles (large, medium or small), one homogeneous soil containing an even mixture of large and medium particles, and two patchy soils consisting of separate patches of large and medium particles and differing in patch scale (large and small patches). We asked the following questions. First, does soil particle size affect the growth (biomass, number of ramets, rhizome length and number of tubers) of *B. planiculmis*, as shown by comparing the treatments with only large, medium and small particles? Second, does patchiness or scale of patchiness of particle size affect the growth of *B. planiculmis* at the level of the whole clone, as shown by comparing the treatments with the homogeneously mixed and the two patchy large and medium particles? Based on effects of heterogeneity of other environmental factors on clonal growth, we expected that the growth in the homogeneously mixed treatment would be lowest and that the growth would differ between the two patchy treatments. Scaling theory predicts that organisms respond to spatial heterogeneity at scales related to their own size [16]. We therefore expected that *B. planiculmis* would show larger responses to soil particle heterogeneity at the scales closer to its spacer length (i.e. inter-ramet distance). Third, does patchiness or scale of patchiness of particle size affect the growth of *B. planiculmis* at the level of the patches? We expected that, in the patchy treatments, the growth of *B. planiculmis* would differ between patch types and that these differences would depend upon patch size.

## Materials and Methods

### Species and Sampling

*Bolboschoenus planiculmis* (F. Schmidt) T. V. Egorova (Cyperaceae), formerly called *Scirpus planiculmis* Fr. Schmidt, is a rhizomatous, perennial, herbaceous, wetland plant [48]. The species is native in China (e.g. in Beijing), and also occurs in India, Japan, Kazakhstan, Korea, Kyrgyzstan, Mongolia, Papua New Guinea, Russia, Tajikistan, southwestern Asia and Europe [48]. It attains heights of 0.6 to 1 m and forms solitary shoots from globose tubers. Shoots die back at the end of one growing season, but tubers can form new shoots and rhizomes that produce new tubers in the next growing season. Both tubers and rhizomes that connect them can survive for several years.

On 10 May 2011, more than 200 ramets, each consisting of a green shoot, a tuber at the base of the shoot and some roots, were collected from a natural population of *B. planiculmis* on the bank of the Beisha River in Beijing. Although the genetic background of these ramets is unknown, they were likely to originate from more than one genet because the longest distance between the sampled ramets was over 50 m. The sampling site did not belong to any farms or national parks and also did not involve any endangered or protected species, so we did not need any relevant permission for collecting plant samples. Each ramet was transplanted into a pot with sufficient nutrition to grow for 15 days. Then 48 ramets of similar size (10–15 cm high with 3–5 leaves) were chosen and used for the experiment.

### Experimental Design

**Figure 1:**
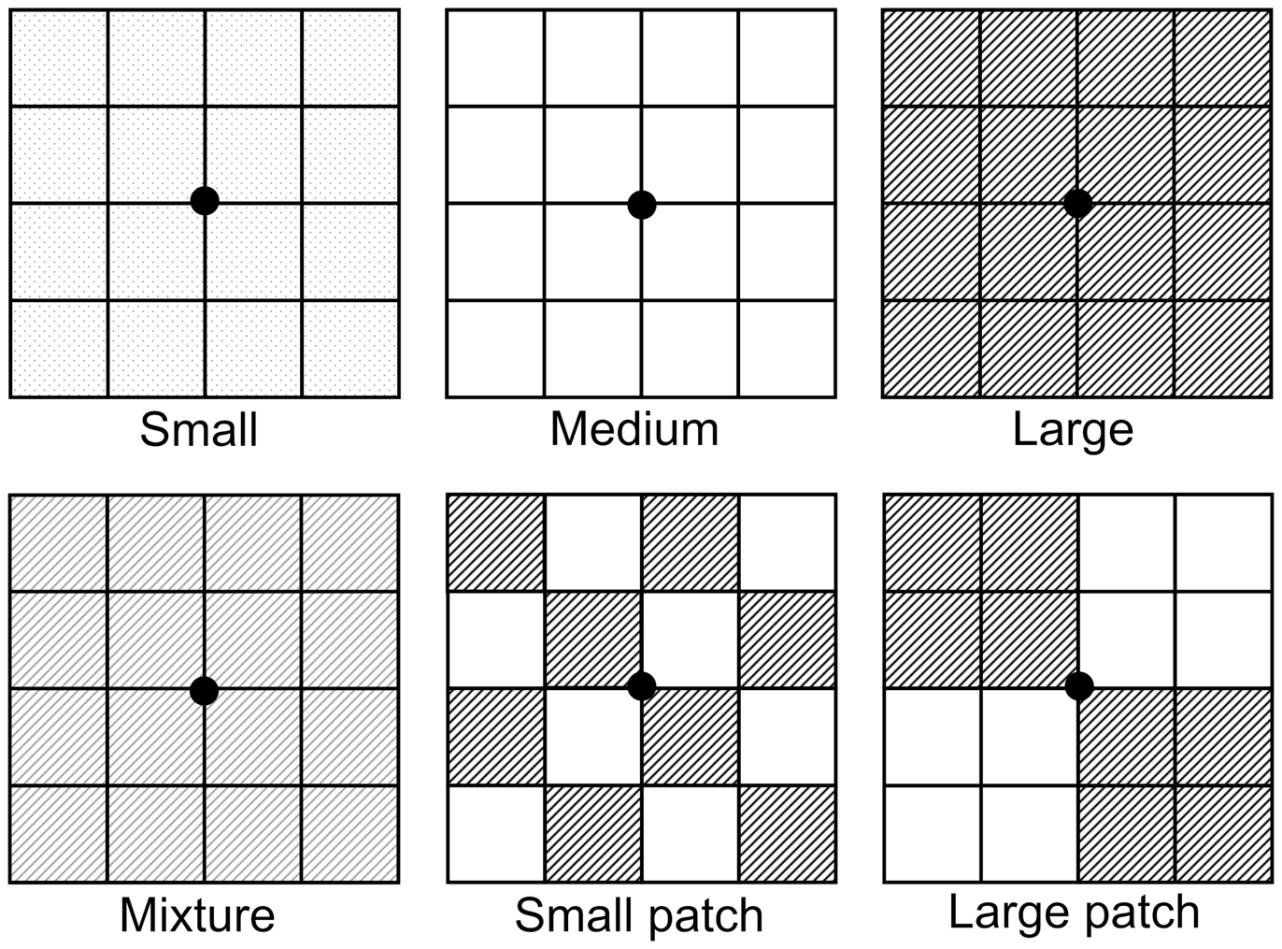
Schematic representation of the experimental design. The experiment had three homogeneous soil treatments with whole boxes filled with small (0.75 mm), medium (1.5 mm) or large (3 mm) quartz (coded as Small, Medium and Large, respectively), one homogeneous treatment with boxes filled with an even mixture of the same amount of large and medium quartz (coded as Mixture), and two heterogeneous treatments consisting of 16 or 4 patches of large and medium quartz (coded as Small patch and Large patch, respectively).

The experiment had three homogeneous treatments with a single type of particle size (termed “Small”, “Medium” and “Large”, respectively), two patchy treatments with different patch sizes (“Large patch” and “Small patch”) and a mixture treatment (“Mixture”; Fig. 1). The substratum used in this experiment was pure quartz without any nutrients. In the Small, Medium and Large treatments, the particle sizes of the quartz were on average 0.75, 1.5 and 3.0 mm, respectively.

In a previous, eight-week, pilot experiment using the Small, Medium and Large treatments, it was found that *B. planiculmis* grew best in the Medium and worst in the Large treatment [49]. The medium and large particle sizes were, therefore, used in the Mixture and patchy treatments. In the Large patch treatment, each box (26 cm long×26 cm wide×13.0 cm deep) was divided equally into four parts (each 13 cm×13 cm), and two parts were filled with medium and two with large particles, in checkerboard fashion (Fig. 1). The Small patch treatment was similar except that each box was divided equally into 16 parts (each 6.5 cm×6.5 cm). In the Mixture treatment, each box was filled with a 1∶1 (v: v) mixture of the medium and large particles (Fig. 1). There were no physical barriers between patches, and roots and rhizomes could grow freely between them. Each treatment had eight replicates (boxes) and there were a total of 48 boxes.

On 5 May 2011, the 48 ramets of *B. planiculmis* were randomly assigned to the six treatments and one ramet was planted in the center of each of the 48 boxes (Fig. 1). The boxes were closed at the bottom so that water and nutrients could not leak. We added 200 ml nutrient solution (1 g Peters Professional [Scotts Professional Limited, Nottingham, UK; N-P-K, 20-20-20] L^−1^ tap water) evenly to each box every week in the first eight weeks. The total amount of nutrients added corresponded to 14.201 g m^−2^ yr^−1^ of each of nitrogen, potassium and phosphorus. The concentration of the nutrient solution used was among the manufacturer's recommended application rate (Scotts Professional Limited, Nottingham, UK) and the nutrients are sufficient for the growth of the plants. Sufficient water was added to the boxes so that the soil in the boxes was flooded and there was always 1 to 3 cm deep water above the soil surface. These measures were to simulate typical wetland conditions experienced by *B. planiculmis* and also ensured that nutrients were evenly distributed in the boxes.

The experiment was conducted in a greenhouse at Forest Science Co., Ltd., of Beijing Forestry University, maintained at a mean temperature of 26.8°C and a mean relative humidity of 72% (measured by iButton DS 1923, Maxim Integrated Products, USA). The boxes were randomly positioned within an area of 18 m^2^ and re-positioned systematically every two to three weeks to minimize effects of position.

### Harvest and Measurements

The experiment lasted 16 weeks and was ended on 14 September 2011. One plant in each of the Small, Small patch and Large patch treatments died during the experiment, and these plants were excluded from harvest and subsequent analyses. In the homogeneous treatments (Small, Medium, Large and Mixture), the mother and offspring ramets were harvested separately. In the two heterogeneous treatments, the mother ramet and the offspring ramets in each type of patches were harvested separately. We counted number of ramets, number of non-sprouted tubers and measured rhizome length. These three variables are commonly used to measure the capacity of clonal growth, and tubers represent potential ramets. Roots, tubers, rhizomes, leaf blades and leaf sheaths were then separated, oven-dried at 70°C for at least 48 h, and weighed.

### Data Analysis

We used one-way ANOVA to examine the effects of soil particle size on the growth (biomass, number of ramets, rhizome length and number of tubers), root-shoot ratio and spacer length (the distance between adjacent ramets) at the whole plant level. In these analyses, we included only the three homogeneous treatments with a single type of particle size (Small, Medium and Large). If a significant effect was detected, a Duncan test was used to compare individual means. We conducted one-way ANOVAs or non-parametric Kruskal-Wallis tests to examine the effects of spatial heterogeneity of soil particles on the growth, root-shoot ratio and spacer length at the whole plant level. In these analyses, three treatments (Small patch, Large patch and Mixture) were used.

We employed two-way ANOVA to examine the effects of particle size (large vs. medium) within patches and patchiness (Large patch vs. Small patch vs. the hypothesized “Null” treatment, see explanation below) on the growth of the plants at the patch level. Data from mother ramets were excluded because they were not located at any patches. The growth pattern (distribution of biomass, ramets, rhizomes and tubers in the two types of soil particle patches) in the Null treatment represented the expected (null) growth pattern in soil without a real patch texture. For this treatment, half values of the corresponding growth variables in the treatment Large were used to represent the expected growth in the large particle patches, and half values of the corresponding growth variables in Medium were used to represent the expected growth in the medium particle patches. We used half values because in the Large patch and the Small patch treatment the plants in each type of patches occupied only half of the area of the whole box. The growth pattern in the Null treatment was used as a null model to show what the pattern was if the placement of biomass, ramets, rhizomes or tubers did not show selectivity (response). For the two heterogeneous treatments, if the growth pattern deviated significantly from the pattern of the Null treatment, as indicated by significant interaction effects of particle size and patchiness, then the plants showed significant selectivity in response to soil particle heterogeneity.

Before analysis, all data were checked for normality (by Shapiro-Wilk test) and homogeneity of variance (by Levene’s test). When we tested the effects of particle heterogeneity on biomass and ramet number at the whole plant level, data could not meet the assumption of normality and/or homogeneity of variance even after transformation. Therefore, non-parametric tests were used for these analyses. Statistical analyses were conducted with SPSS 18.0 for Windows (SPSS, Chicago, IL, USA).

## Results

### Effects of Soil Particle Size at the Whole Plant Level

**Figure 2:**
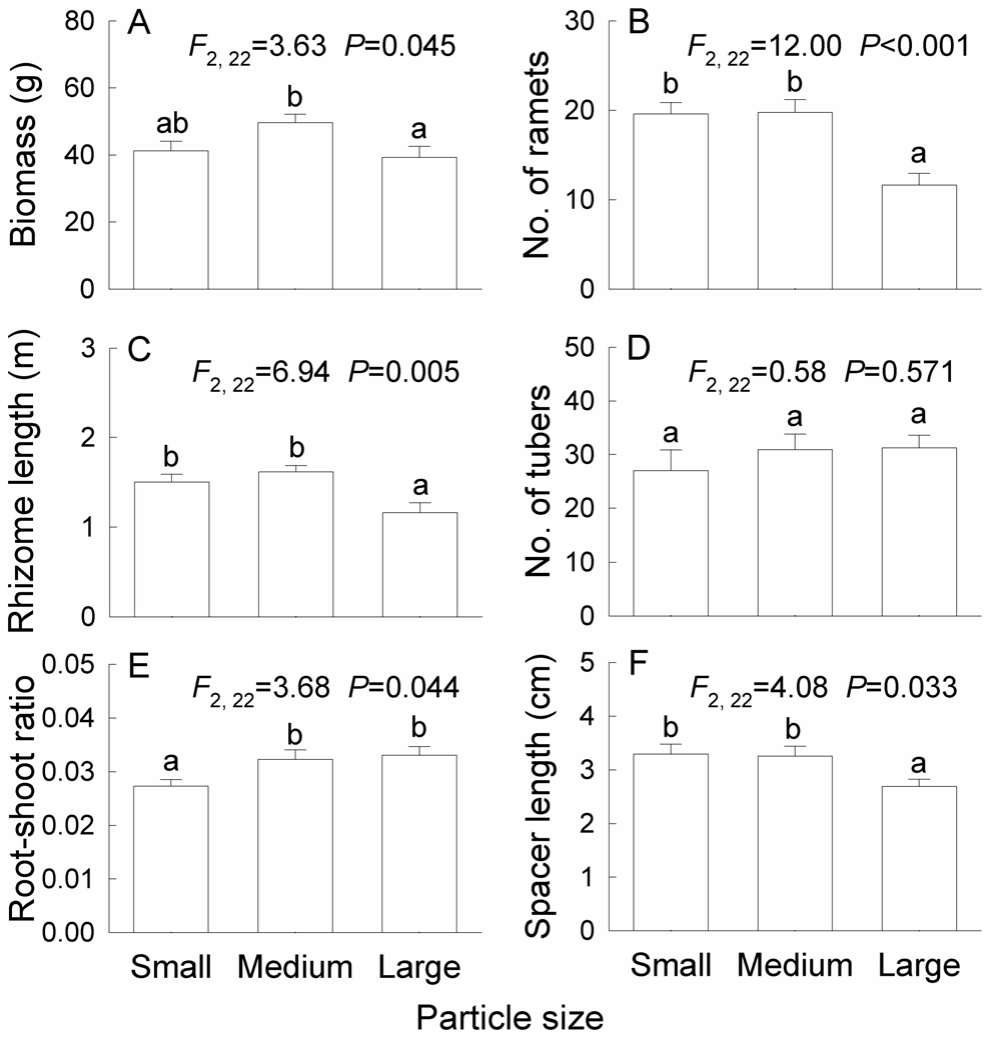
Effects of soil particle size on growth (A–D) and morphology (E, F) in the treatments with a single particle size. The treatment codes are described as in Fig. 1. Bars and vertical lines are means and SE. Bars sharing the same letters are not different at *P* = 0.05.

In the homogeneous treatments, biomass, number of ramets, rhizome length and spacer length of *B. planiculmis* were significantly greater in the Medium than in the Large treatment, but they did not differ between the Small and Medium treatments (Fig. 2A–C and F). Root-shoot ratio was higher in the Medium and Large treatments than in the Small treatments (Fig. 2E). Soil particle size did not affect number of tubers (Fig. 2D).

### Effects of Soil Particle Heterogeneity at the Whole Plant Level

None of the growth or morphological measures of the whole plants differed significantly between the Mixture, Small patch and Large patch treatments (χ^2^ = 2.47–3.16, *P*  = 0.206–0.290 for biomass and number of ramets; *F*_2, 21_ = 0.501–2.186, *P*  = 0.140–0.614 for rhizome length, number of tubers, spacer length and root-shoot ratio). The result suggests that soil particle heterogeneity did not affect the growth or morphology at the whole plant level.

### Effects of Soil Particle Heterogeneity at the Patch Level

**Figure 3:**
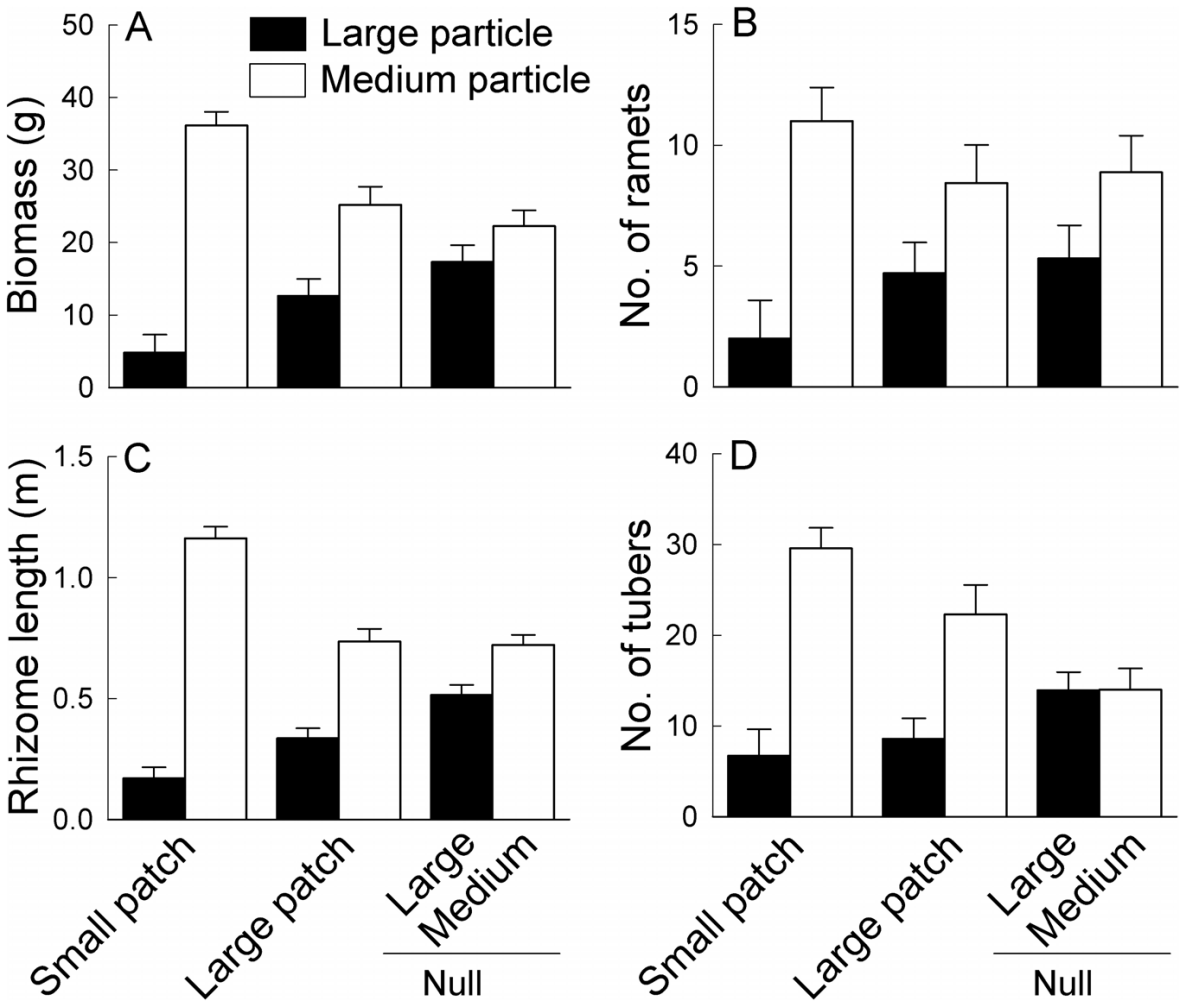
Effects of patchiness and particle size within patches on the growth within patch types. The treatment codes are described as in Fig. 1. In the “Null” treatment, half values of the corresponding variables in Large and Medium were used because in Large patch and Small patch the plants in each type of patches occupied only half of the area of the whole box. Bars and vertical lines are means and SE.

At the patch level, particle size significantly affected biomass, number of ramets, rhizome length and number of tubers of *B. planiculmis* (Table 1). In general, biomass, number of ramets, rhizome length and number of tubers were greater in the medium than in the large particle patches (Fig. 3), and such effects were the largest in the Small patch and smallest in the Null treatment (Table 1: significant interaction effect of particle size and patchiness; Fig. 3).

**Table 1:** Effects of particle size within patches and patchiness on the growth at the patch level.

Effects	DF	Biomass	No. oframets	Rhizomelength	No. oftubers
Particle size	1,38	100.5***	70.0***	71.3***	35.5***
Patchiness	2,38	0.3^ns^	0.3^ns^	1.4^ns^	1.4^ns^
Interaction	2,38	23.3***	7.5**	14.0***	10.8***

F-values and degree of freedom (DF) of two-way ANOVAs are given. *Superscripts give P:* ***P<0.001, **P<0.01 and ^ns^ *P*≥0.05.

## Discussion

Soil particle size significantly affected the growth of *B. planiculmis*: plants accumulated more ramets and biomass in soil composed of medium (diameter 1.5 mm) or small (0.75 mm) particles than of large (3.0 mm) particles. This result suggests that differences in soil particle size should be considered when we use different soil types to test the effects of soil nutrient availability. Soil particle size may greatly affect both chemical and physical properties of soil [33], [36], [50]–[51]. In the present study, the effects on soil chemical properties may be of little importance because sufficient nutrient solutions were supplied to each plant during the experiment. Previous studies have showed that changing soil physical properties such as soil mechanical resistance, infiltration capacity and adsorption capacity can significantly influence the growth and reproduction of plants [51]–[52]. For example, increasing soil mechanical resistance may slow down root growth and root expansion [52]–[56]. In the present study, each plant was supplied with the same, sufficient amount of nutrients and also grown in simulated wetland (flooded) conditions. Therefore, changing soil infiltration capacity or adsorption capacity caused by different soil particle sizes may also be of little importance. One likely explanation is that differences in soil mechanical resistance mediated by different soil particle sizes are responsible for the difference in the biomass production and clonal reproduction of *B. planiculmis*. Compared to soil composed of small or medium particles, soil composed of large particles is likely to have a higher mechanical resistance, which significantly decreased the growth of *B. planiculmis*. Further studies are required to test the relationships between soil particle size and soil mechanical resistance.

Soil particle heterogeneity significantly affected the growth of *B. planiculmis* at the patch level. Compared to the homogeneous large particle treatment, *B. planiculmis* produced less biomass and tubers and shorter rhizomes in the comparable area in the large particle patches in the two heterogeneous treatments, and such effects were greater in the small patch than in the large patch treatment. Also, compared to the homogeneous medium particle treatment, *B. planiculmis* produced more biomass and ramets and longer rhizomes in the comparable area in the medium particle patches in the small patch treatment, but not in the large patch treatment. As a result, in the heterogeneous treatments, comparably more ramets, tubers, biomass and rhizomes were placed in the patches composed of medium particles, which are more favorable for the growth of *B. planiculmis* [49]. These responses are similar to the foraging responses of clonal plants when growing in resource-heterogeneous environments where clonal plants commonly position more ramets in favorable (resource-rich) patches and less in unfavorable (resource-poor) patches [21], [24], [41], [57]–[58]. Such responses are thought to be adaptive since they potentially can increase resource uptake of the whole clone [57], [59]–[62].

However, at the whole (clone) plant level, we found that soil particle heterogeneity did not affect the growth of *B. planiculmis*. In contrast, spatial heterogeneity consisting of patches of fertile and infertile soil could greatly increase the whole plant growth of some clonal plants such as *Glechoma hederacea* and *Alternanthera philoxeroides* [40]–[41], [63]. Therefore, in these studies the effects of soil heterogeneity at the whole plant levels are very likely due to soil nutrient heterogeneity. When more ramets are located in nutrient-rich patches, both the efficiency and the total amount of nutrient uptake increase markedly [41]. This will result in a significant increase in nutrient uptake of the whole clone as well as its subsequent growth. In the present study, however, because differences in soil mechanical resistance might be the main mechanism underlying the effects of soil particle heterogeneity, the patch-level effects on the selectivity of where ramets are placed and their growth in the patches could not translate into the effects at the whole clone level.

The effects of soil particle heterogeneity were greater in the small patch than in the large patch treatment, suggesting that patch scale may have played an important role during the responses of *B. planiculmis* to soil particle heterogeneity. Previous studies have also shown that patch scale affected the responses of *Glechoma hederacea* [20]–[21] and *Colophospermum mopane* [16] to soil heterogeneity and *Duchesnea indica* to light heterogeneity [19]. In the present study, the smaller the patch scale was, the more the ramets were placed in the medium particle patches than in the large particle ones. The larger effects of soil particle heterogeneity at the smaller scale were likely because the mean inter-ramet distance (spacer length, 2.3–3.7 cm on average, Fig. 2F) of *B. planiculmis*, which partly determines the placement and density of ramets in a patch [57], [60], [64], was closer to the patch size (6.5 cm×6.5 cm) in the small patch treatment. This result agrees with scaling theory, which predicts that organisms respond to spatial heterogeneity at scales related to their own size [16], [65]. Another possible interpretation is that in the small patch treatment plants had a higher probability of finding a favorable patch (i.e. consisting of medium particles) when such favorable patches were more spread out. However, this interpretation should be considered only when patch sizes are within a range that plants can sense them, such as the two patch sizes used in the present study. If patch size is set smaller than this range, plants will likely not be able to respond to spatial heterogeneity [65].

In conclusion, soil particle size and heterogeneity may greatly affect the growth of plants. Thus, when we address the effects of soil nutrient availability or the effects of soil nutrient heterogeneity, caution should be taken when different soil types are used to form low and high nutrient conditions, because different soil types may differ greatly in soil particle composition [31]–[32], [66]–[67]. Further studies testing the effects of soil heterogeneity should distinguish the effects of soil nutrient heterogeneity from those of soil particle heterogeneity. The results also suggest that scale should always be considered when we address the effects of spatial heterogeneity [16], [18]–[21].
